# Thermal Conductivity of CdCr_2_Se_4_ Ferromagnet at Low Temperatures: Role of Grain Boundaries and Porosity

**DOI:** 10.1021/acs.langmuir.5c06870

**Published:** 2026-03-18

**Authors:** Jiří Hejtmánek, Kyo-Hoon Ahn, Zdeněk Jirák, Petr Levinský, Jiří Navrátil, Sandy Al Bacha, Emmanuel Guilmeau, Karel Knížek

**Affiliations:** † Institute of Physics of the Czech Academy of Sciences, Prague 6 162 00, Czech Republic; ‡ CRISMAT, CNRS, 131990 Normandie Univ, ENSICAEN, UNICAEN, Caen 14000, France

## Abstract

It is unambiguously demonstrated that the low-temperature magnon specific heat in a ferromagnet varies as *T* ^3/2^ and the magnon thermal conductivity, due to the *T* ^1/2^-dependent effective velocity of magnons, varies as *T* ^2^. The confirmation of these model behaviors is based on the experimental study of chalcospinel CdCr_2_Se_4_, which represents a relatively rare example of a ferromagnetic insulator (*T*_C_ = 130 K) without undesirable “masking” contributions from itinerant electron excitations and nuclear specific heat, both of which make it impossible to conclusively unveil the role of magnons. The ratio of the magnon to lattice specific heat is found to reach 87:13 at 2 K and is in accordance with predictions based on the spin-wave stiffness *D* = 33.5 meV Å^2^ and Debye temperature θ_*D*_ = 237 K. On the other hand, the ratio of the magnon to phonon thermal conductivity, reaching 27:73 at 2 K, is much lower than expected for the standard model of grain boundary-limited transport. This suggests that mean free paths for long-wavelength magnon/phonon heat carriers are largely different—shorter than the grain size (∼1 μm) for magnons and longer than the grain size for phonons. The phonon-dominated low-temperature thermal conductivity, moreover, exhibits a ∼*T* ^2.3^ temperature dependence instead of the standard predicted model of *T* ^3^. The relevant scattering mechanisms, both the phonon frequency-independent and frequency-dependent ones, are discussed in detail.

## Graphical abstract

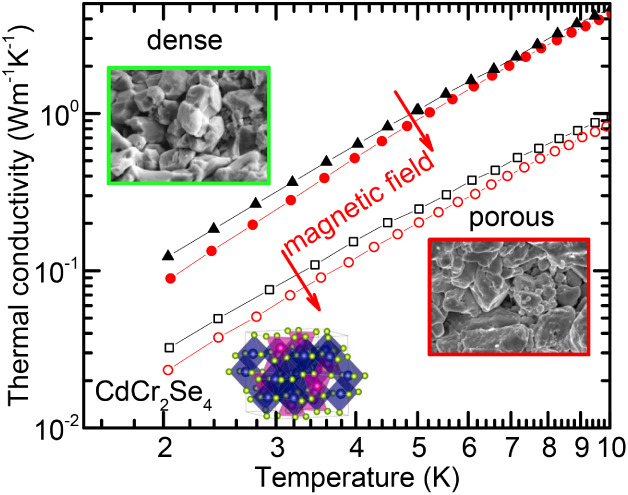

## Introduction

Although the low-temperature thermal properties of ferromagnets are routinely interpreted using textbook theoretical models, the contribution of spin-wave excitations (magnons) is difficult to clearly assess and analyze experimentally, namely due to the more significant contribution of lattice excitations (phonons). Phonon and magnon contributions become clearly distinguishable only at very low temperatures where, unfortunately, they are often masked by other pertinent contributions: namely, those of electronic origin (itinerant electrons in metallic systems), (ii) Schottky type (both electronic and nuclear), or (iii) “extrinsic-like” associated with phase purity or sample morphology (e.g., a glassy state artificially boosting the low-temperature heat capacity). In this report, we have chosen as a model compound the insulating ferromagnetic selenospinel CdCr_2_Se_4_ (*T*_C_ ∼ 130 K), in which all the above-mentioned inconveniences are eliminated.

The CdCr_2_Se_4_ phase is characterized by a spinel structure of cubic *Fd*3̅*m* symmetry, with the nonmagnetic Cd^2+^ cations in tetrahedral sites and magnetic Cr^3+^ cations in octahedral sites. In such a case, there are generally two competing interactions between nearest-neighbor transition cations: the antiferromagnetic direct 3d−3d exchange and the 90° ferromagnetic superexchange via anionic p-orbitals, each with specific dependence on orbital overlaps.^1–3^ While closely related oxospinels ZnCr_2_O_4_ or CdCr_2_O_4_ are frustrated antiferromagnets, the present system, possessing large Se^2−^ ions and reduced direct exchange, is a stable ferromagnetic insulator.^4,5^ Concerning the thermal properties, it should be stressed that the selected compound does not contain any element possessing noticeable low-temperature nuclear *C*_*p*_ contribution nor electronic Schottky contribution (the $t_{2g}^{3}$ orbital singlet state of Cr^3+^ yields an ideal spin moment of 3 μ_B_/Cr and lacks any close-energy excited states). Finally, the risk of impurities or glassy phase formation is minimized, as the phase diagram enables the synthesis of a pure phase with minimum contamination by impurities. Moreover, the cubic symmetry, moderate Curie temperature, and absence of elements with significant spin−orbit coupling anticipate the possibility of suppressing magnons by an external magnetic field. We note that, in the case of other well-known Ferromagnetic insulators, like EuO or others based on rare-earth metals, the noticeable nuclear contribution to the specific heat below 1 K makes the analysis of magnons difficult.^6^

As the CdCr_2_Se_4_ system and its electronic and magnetic properties are concerned, the detailed theoretical survey, *ab initio* calculations and basic experimental characterization have been presented in our previous publication.^7^ Here, we focus on the low-temperature specific heat capacity and thermal conductivity of ceramic CdCr_2_Se_4_ samples and exemplify the suppression of respective magnon-controlled contributions by the application of a strong magnetic field. Due to the insulating character of CdCr_2_Se_4_, the specific heat capacity is composed of only two terms: the contribution of transversal and longitudinal acoustic lattice modes and the contribution of a single acoustic mode of spin-wave excitations. The same bosonic particles (phonons and magnons) contribute as heat carriers to the thermal conductivity. We examine especially the effects of grain boundaries and sample porosity that are decisive for the thermal transport of polycrystalline CdCr_2_Se_4_ below 10 K.

## Experimental

Ceramic samples of CdCr_2_Se_4_ were prepared from a stoichiometric mixture of an intimate combination of pure elements. The mixture was heated in an evacuated silica ampule up to 1073 K, then quenched into cold water, reground, and heated again in an evacuated ampule up to 973 K for another 5 days. The final powdered product was sintered using either

I. Hot-pressing at 763 K and a pressure of 50 MPa for 1 h resulted in relatively porous ceramics with a relative density of 83%. The sample is further labeled as porous.
II. Spark plasma sintering (SPS), where the powder was placed into tungsten carbide dies and densified using SPS (SPS-FCT HPD 25) at 773 K for 30 min under a pressure of 300 MPa (24 kN), produced pellets with a 10 mm diameter and a thickness of ∼6.5 mm, with geometric densities exceeding 95%. Considering the extreme sintering conditions, the sintering was followed by annealing at 500 °C in a selenium atmosphere for 24 h. The sample is further labeled as dense.

The annealing in a selenium atmosphere enabled the recovery of the high phase purity of the ceramics, similar to the hot-pressed sample, i.e., with the main impurities identified both by energy-dispersive X-ray spectroscopy (EDS) and X-ray diffraction (XRD) as CdSe and Cr_2_Se_3_ with concentrations below 1% for both (see the Supporting Information). The phase purity of both samples was checked by powder X-ray diffraction acquired on a Bruker D8 Advance powder diffractometer with CuKα radiation, equipped with a Lynxeye XE-T detector. The microstructure of the samples was analyzed by scanning electron microscopy and a microanalyzer Jeol JXA-8230 with EDS Bruker QUANTAX 200 (software Esprit 2.5 ED). The average grain size, larger than 1 μm, deduced from the width of diffraction peaks, agrees with the SEM images showing grains with sizes in the range of units of μm (see the Supporting Information). The EDS analysis of the dense ceramics revealed an average composition of Cd_1.01(0.02)_Cr_2.02(0.06)_Se_3.97(0.04)_, which is very close to the ideal spinel stoichiometry (see Table S1).

The magnetic response was measured using a superconducting quantum interference device (SQUID) magnetometer (MPMS-XL, Quantum Design); the thermal properties were characterized by means of the Physical Property Measurement System (PPMS, Quantum Design). For a more detailed description, please see ref.^7^.

## Results and Discussion

### Fundamentals of Polycrystalline CdCr_2_Se_4_: Magnetism and Thermal Properties

Ferromagnetic ordering of the studied CdCr_2_Se_4_ is demonstrated in Figure 1. The negligible difference between the FC (field-cooled) and ZFC (zero-field-cooled) magnetic susceptibility confirms the good magnetic homogeneity, without any tendency to form clusters or inhomogeneities. The ideal ferromagnetic state is corroborated by the magnetic moment corresponding to 3 μ_B_/Cr, whose value perfectly matches the spin-only contribution of octahedrally coordinated Cr^3+^ ions.

**Figure 1:**
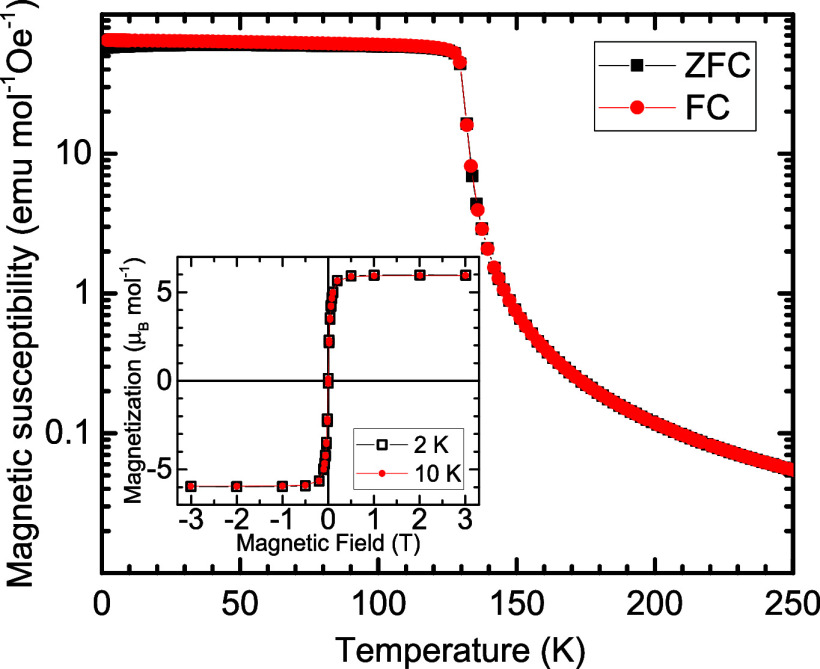
Temperature dependence of the magnetic susceptibility of CdCr_2_Se_4_ measured at a DC field of 100 Oe. In the inset, the magnetization reaches 6 μ**_B_**/f.u., confirming an ideal ferromagnetic state at low temperatures.

The temperature dependence of the specific heat capacity is shown in Figure 2. The ferromagnetic transition is characterized by a small peak at 130 K; the faint feature detected at *T*_C_ corroborates its second-order character. The logarithmic scale of Figure 2 enables us to visualize the low-temperature behavior, where the comparison between the data measured at 0 and 13 T clearly uncovers the huge impact of the magnetic field on the heat capacity. This is further highlighted in the inset, where the magnetic field dependence of the heat capacity at 2 K shows a gradual suppression of the specific heat and its final saturation under 13 T at only ∼13% of its original value. Consequently, the specific heat measured within the range 0.35−5 K displays, under zero magnetic field, the temperature dependence *C*_*p*_ ∼ *T* ^3/2^, which perfectly agrees with theoretical predictions for a simple cubic ferromagnet magnon-specific heat, and changes under strong magnetic field to the classic phonon-dominated dependence *C*_*p*_ ∼ *T* ^3^ (see the main panel of Figure 2). To better visualize the relative impact of lattice and spin waves at low temperatures, we depict the low-temperature specific heat capacity in a standard way as *C_p_*/*T* vs. *T* ^2^ in Figure 3.

**Figure 2:**
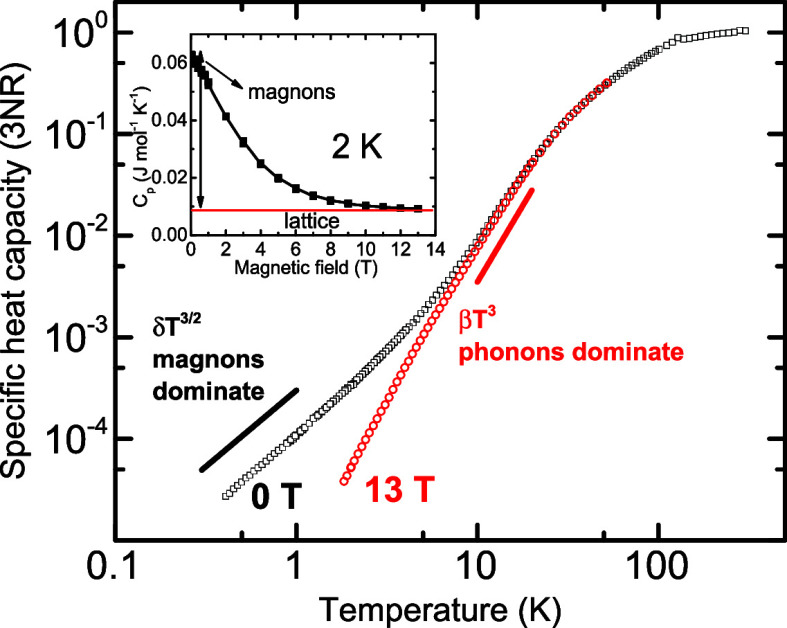
Temperature dependence of CdCr_2_Se_4_ specific heat capacity expressed as a relative value with respect to the Dulong−Petit limit. The data at zero field (black) and μ_0_ *H* = 13 T (red) are shown. In the inset, the field-induced suppression of the magnon contribution to the specific heat is evidenced at 2 K, where the magnetic contribution represents 87% of the total heat capacity.

**Figure 3:**
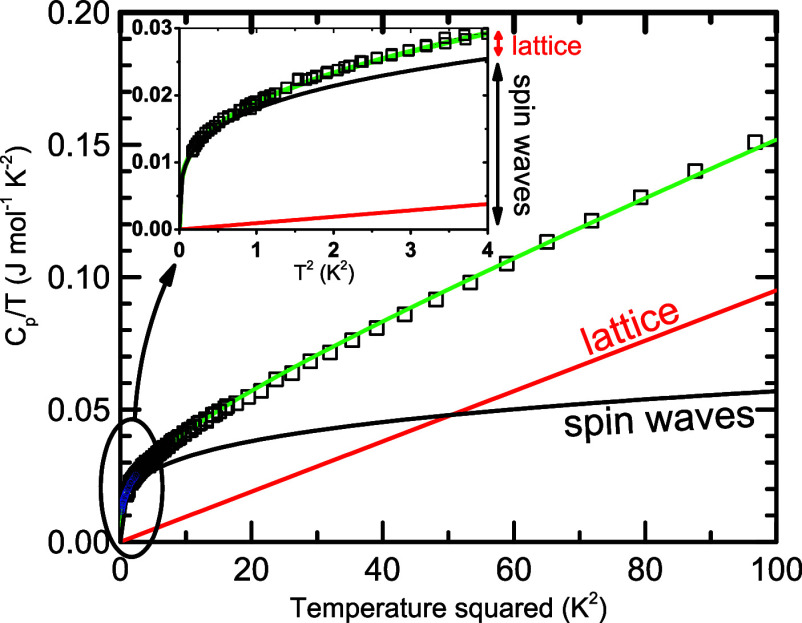
Low-temperature specific heat capacity of CdCr_2_Se_4_ expressed in a standard way. The splitting between the lattice (red line) and magnon (black line) contributions provides a perfect agreement between theoretical expectations (green line) and experiments (open squares). In the inset, the experimental data down to 350 mK confirm the dominating contribution of magnons to specific heat capacity below 2 K; the missing low-temperature rise of specific heat corroborates the absence of nuclear magnetic moments of stable isotopes of Cd, Cr, and Se.

Low-temperature behavior of CdCr_2_Se_4_ agrees with the quantification of the specific heat capacity in cubic ferromagnetic insulators as a sum of the lattice and magnon terms. Molar lattice heat is given by Debye formula

1 $$C_{ph}\left(T\right)=\frac{12\pi^{4}}{5}nN_{A}k_{B}{\left(\frac{T}{\Theta_{D}}\right)}^{3}$$

where *n* is the number of atoms in the formula unit (*n* = 7 for CdCr_2_Se_4_), *N*_A_ is Avogadro’s number, and θ_*D*_ is the Debye temperature. The molar magnon heat is derived for the case of a simple, gapless quadratic dispersion for spin waves, *E* = ℏ*ω_k_* = *Dk* ^2^, and is applicable at zero magnetic field only,

2 $$C_{m}\left(T\right)=0.113V_{mol}k_{B}{\left(\frac{k_{B}T}{D}\right)}^{3/2}$$

where *V*_*mol*_ is the molar volume (72.4 × 10^−6^ m^3^ for CdCr_2_Se_4_) and *D* is the spin-wave stiffness (see, e.g., refs. 8–10 ). Under a magnetic field, the magnons are gradually depopulated because of the Zeeman gap △ = *g_J_μ_B_μ_o_H* for magnon excitations. Here, *H* is the applied field corrected for demagnetization fields (for details, see the macroscopic magnon theory on p. 63 of ref.^8^) *μ*_0_ is the permeability of vacuum, *μ_B_* is the Bohr magneton, and *g_J_* ∼ 2 for transition metal ions with frozen orbital momenta. The gap brings about a gradual suppression of the magnon heat capacity that scales as *H*/*T*. The calculated “quenching” curve can be found elsewhere;^7,9^ it matches rather well with the experimental curve seen in the inset of Figure 2.

Confirming that the low-temperature specific heat of CdCr_2_Se_4_ perfectly fits the theoretical models, we evaluate *C_tot_* (*T*) = *βT* ^3^ + *δT* ^3/2^ with the phonon term determined by β = 1.02 × 10^−3^ J K^−4^ mol^−1^ and magnon term given by δ = 19 × 10^−3^ J K^−2.5^ mol^−1^. The value of β corresponds to the Debye temperature θ_*D*_ = 237 K, which falls between 180 K obtained in ref.^11^ and 280 K in ref.^12^. Considering the quadratic magnon dispersion, the value of δ corresponds to the spin-wave stiffness *D* = 33.5 meV Å^2^, which is in perfect agreement with the values obtained from the spin-wave resonance technique (*D* = 31.3 meV Å^2^ ^13^) and previous heat capacity measurements (*D* = 33 meV Å^2^ ^12^).

We are now turning to the thermal conductivity data of CdCr_2_Se_4_ displayed as a log−log graph in Figure 4 (for a normal scale, see Figure 5a below). At ∼50 K, the thermal conductivity develops a broad maximum, reminiscent of a typical phonon peak that arises in single-crystal materials with increasing temperature as a compromise between enlarged phonon population and more frequent phonon−phonon scattering. The thermal conductivity maximum appearing at ∼θ_*D*_/5 is of only a moderate value, which points to the heat transport limitations due to the polycrystalline character of the sample and its porosity. At higher temperatures (above ∼Θ_*D*_/2), it is generally considered that the mean free path of heat-carrying phonons is essentially influenced by dissipative phonon−phonon Umklapp processes. Based essentially on the simple model of acoustic phonons, the thermal conductivity is expected to vary as *T* ^−1^. In reality, one should consider different dispersion relations of the longitudinal/transversal phonons and the presence of optical phonons, which often have only a minor contribution to heat transport but provide significant scattering channels for the acoustic phonon transport (for more information, see, e.g., refs. 14,15 ). The *ab initio* theoretical calculations of intrinsic thermal conductivity have been recently performed for Si with a diamond structure^16^ and CrN with a distorted rock-salt structure,^17^ both having 3 optical phonon branches, or for a more complex case of the defect-chalcopyrite CdGa_2_Se_4_ with 18 optical branches.^18^ Remarkably, all of these calculations show the high-temperature thermal conductivity to follow very closely the ideal *T* ^−1^ trend. As far as the present CdCr_2_Se_4_ system is concerned, its spinel structure gives rise to phonon spectra with 3 acoustic and 39 optical branches,^7^ making eventual *ab initio* calculation of phonon−phonon processes extremely tedious. Moreover, the thermal conductivity in Figure 4 follows in the paramagnetic state, a different trend, namely the *T* ^−1/2^ dependence, which we tentatively associate with local spin correlations and interconnected atomic displacements that act as phonon scatterers above the Curie temperature (*T*_C_ ∼ 130 K).^19^ We note, however, that the presence of additional scattering, namely point defect-like scattering, may also cause a similar deviation from *T* ^−1^ to the ∼*T* ^−1/2^ temperature dependence, as shown already by Klemens.^20^ In the case of our sample, we favor the “magnetic” scenario since the deviation from the standard Umklapp-dominated *T* ^−1^ temperature dependence starts sharply at *T*_C_ (see Figures 4 and 5). We present further discussion on this subject at the beginning of the next section.

**Figure 4:**
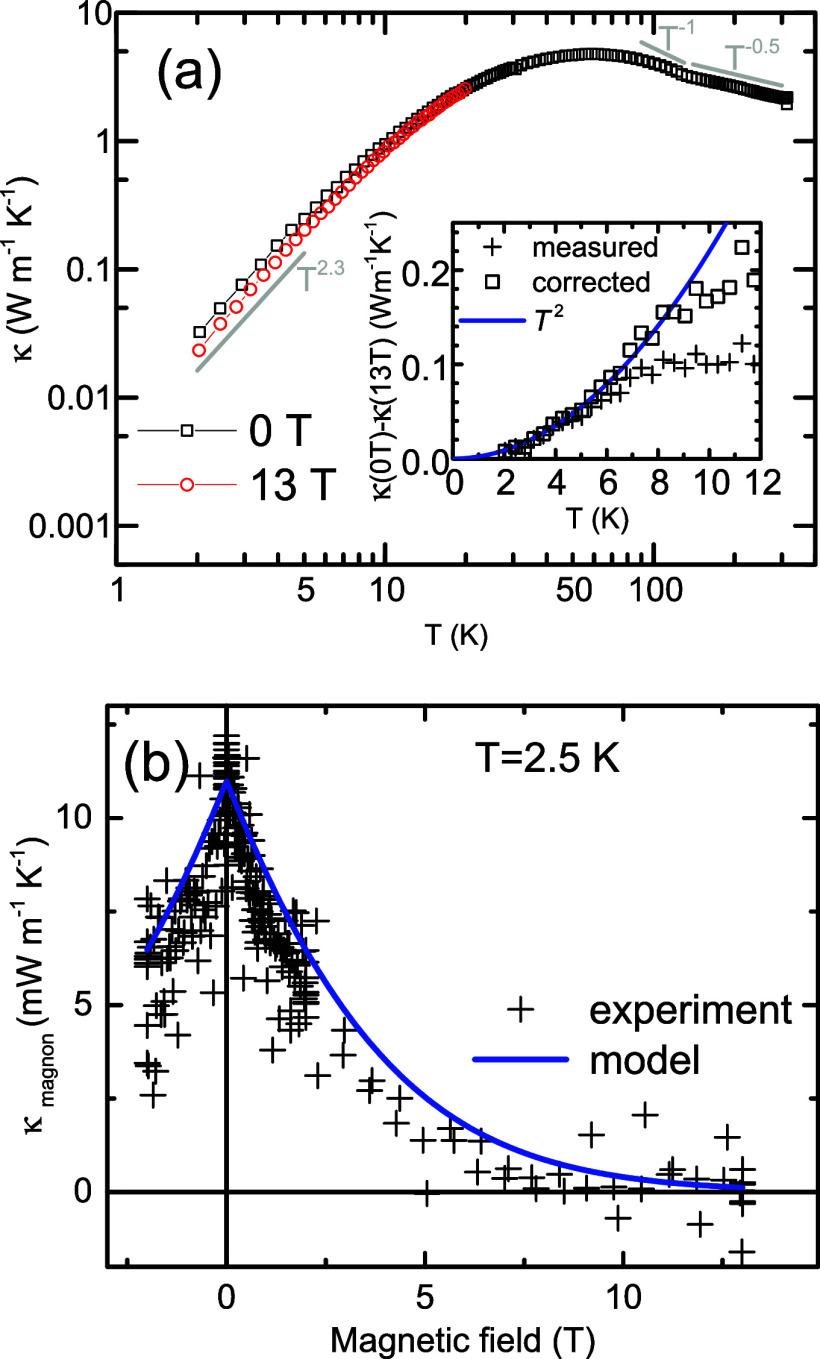
Temperature dependence of the thermal conductivity of CdCr_2_Se_4_ (upper panel a). The ceramic character of the sample (porosity 17%, grain size ∼1 μm) is responsible for low-temperature behavior, which follows the ∼*T* ^2.3^ dependence. In the inset, we depict the magnon contribution to the thermal conductivity κ_m_ ∼ *T* ^2^. The magnon contribution κ_m_ was determined as the difference between κ_0T_ and κ_13T_ (crosses), with additional correction for incomplete quenching of the magnon contribution above 6 K (squares). The lower panel (b) shows the suppression of the magnon thermal conductivity at 2.5 K; complete quenching of magnon thermal conductivity at 13 T is supposed.

**Figure 5:**
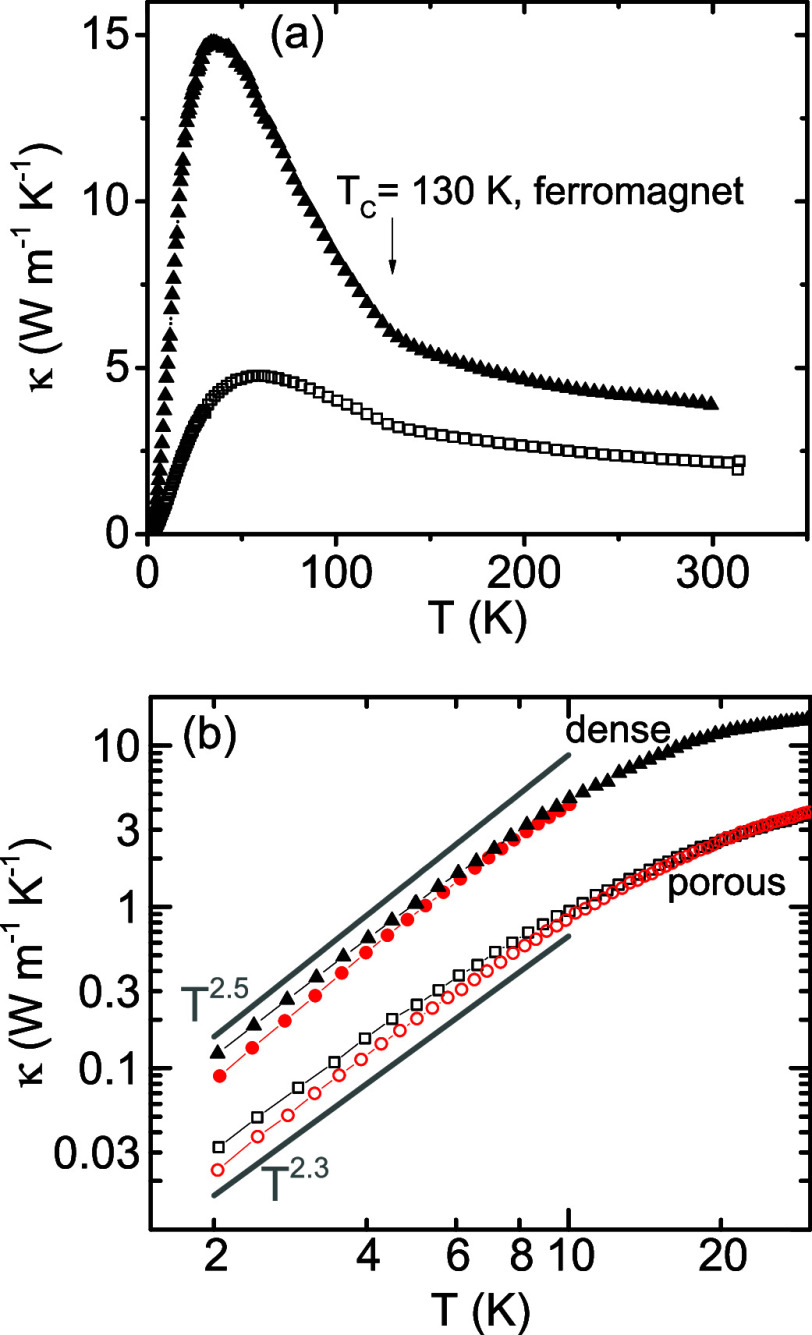
(a) Thermal conductivity of a dense CdCr_2_Se_4_ sample (5% porosity, solid triangles) compared to the original porous one (17% porosity, open squares). (b) The effect of an external field of 13 T at low temperatures (red symbols).

Our primary concern is, however, the behavior of thermal conductivity well below the phonon peak, especially with respect to the phonon/magnon contributions *κ_ph_* and *κ_m_*. As seen in Figure 4, the thermal conductivity follows the *T* ^2.3^ dependence, and only a minor suppression is observed under a field of 13 T. The isothermal field dependence, demonstrating the gradual quenching of *κ_m_*, is shown for 2.5 K in the lower panel (for more data and discussion of *κ_m_* (*H*,*T*), see ref.^7^). The difference between 0 and 13 T is presented in the inset of Figure 4a. It should be noted that the quenching at 13 T becomes incomplete with increasing temperature (e.g., it amounts to 92% at 4 K and 68% at 8 K, respectively).^7,10^ The data, after proper correction, follows the *T* ^2^ dependence rather well up to about 8 K. The deviation observed at higher temperatures will be commented on in more detail at the end of the next chapter; we just note that, instead of the quasi-exponential quenching exemplified for 2.5 K and scaled as *H/T*, there is a certain drop in thermal conductivity in low fields, followed by a nearly constant value in the range of 1−13 T.

In a first approximation, we confront the data with predictions based on the kinetic gas theory.^8,19,21^ Neglecting the role of grain boundaries, such a conventional model gives the low-temperature thermal conductivity of a ferromagnetic insulator as a sum of lattice and magnon terms, $\kappa =\frac{1}{3}C_{ph}v_{ph}l_{ph}+\frac{1}{3}C_{m}v_{m}l_{m}$ . Here, *C*_*ph*_ (*C*_*m*_) is the lattice (magnon) specific heat per unit volume, *v*_*ph*_ (*v*_*m*_) is the sound velocity of acoustic phonons (group velocity of magnons), and *l*_*ph*_ (*l*_*m*_) is the phonon (magnon) mean free path as the main unknown parameter. With decreasing temperature, the *l*_*ph*_ (*l*_*m*_) values gradually increase because of decreased populations of both quasiparticles (acoustic phonons and magnons) and, consequently, fewer collisions among them. In high-quality single crystals, the free path eventually becomes limited by the dimensions of the sample, based on Casimir theory, which states that a nonideal (coarsened) crystal boundary absorbs the heat of impacting quasiparticles and emits a new equilibrium spectrum^22^ (for experimental confirmation, see ref.^23^). Considering the phonon term of thermal conductivity *κ_ph_*, theoretical *κ_ph_* ∼ *T* ^3^ temperature dependence is obtained based on the standard *T* ^3^ variation of lattice heat capacity, constant velocity of acoustic phonons, and phonon-frequency-independent mean free path given by Casimir. Similarly, the low-temperature magnon term is proposed (and indeed observed on CdCr_2_Se_4_) to be *κ_m_* ∼ *T* ^2^, due to the *T* ^3/2^ variation of magnon specific heat and *T* ^1/2^-dependent effective velocity of magnons.

The decline of the CdCr_2_Se_4_ phonon thermal conductivity *κ_ph_* ∼ *T* ^2.3^ from the simple theoretical *T* ^3^ dependence (conditioned by temperature-independent scattering) points to the existence of frequency-dependent scattering mechanisms influencing thermal conductivity down to very low temperatures. First, it can be noted that, even within the Casimir model, the deviation from *T* ^3^ can be obtained, as shown already by Ziman, who considered the possibility of some specular reflection at sample boundaries of given roughness, leading to a frequency-dependent ratio of the diffusive and specular reflection terms. As far as natural defects in crystals are concerned, a very specific behavior of phonon transport has been observed in metals and alloys, namely due to dislocations that are supposed to move in a quasi-free manner under the striction strain of a dynamic elastic wave represented by phonons (the movement called by Ziman as “fluttering”^24^). The energy dissipated is then proportional to the phonon frequency ω, and if dominant, this mechanism naturally leads to the *T* ^2^ variation of the phonon part of the metal thermal conductivity (see also ref.^25^). Recently, Kim et al., in their optimization of the Bi_2_Te_3_-type thermoelectric alloys, demonstrated a technology leading to a “mosaic crystal”, where individual blocks are separated by low-angle boundaries with dense periodic arrays of dislocations. Here again, strong ω-dependent phonon scattering is obtained, but instead of the freely moving dislocations, the mechanism relies on the static strain they impose.^26,27^

Remarkable changes in phonon transport compared to pure crystalline systems were also observed by Pohl and colleagues in numerous alkali halides with complex anionic substituents. For KBr:NO_2_ single crystals in particular, the scattering mechanisms have been identified as rotation, liberation, and tunneling processes at NO_2_^−^ sites, which provide a broad spectrum of low-energy excited levels. Such local excitations are responsible for thermal conductivity being strongly suppressed already from the phonon peak temperature, with apparent *T* ^2^ dependence down to helium temperature. However, below ∼1 K, the thermal conductivity starts to recover.^28^ The present polycrystalline sample of CdCr_2_Se_4_ shows a low-temperature thermal conductivity very similar to that of KBr:NO_2_, both in magnitude and temperature dependence. Scattering mechanisms, however, now originate in grain boundaries and other associated imperfections, which complicate a clear understanding and simple modeling of thermal conductivity. On the other hand, any effort in this direction is highly desirable, since phonon grain boundary scattering can suppress the transmittivity of long-wavelength phonons. In the case of nanosized grains, the thermal conductivity can be largely reduced even at ambient temperatures. Nanostructuring is thus an effective tool for improving the performance of thermoelectric materials.^29,30^

Before presenting relevant experiments on CdCr_2_Se_4_, we consider it important to note some illustrative examples of the combined role of various volume defects associated with the grain boundaries and sample porosity that are projected onto the thermal transport of “real” polycrystalline ceramics.

I. There are some cases where thermal conductivity is largely reduced in polycrystalline materials but still follows the *T* ^3^ dependence at low temperatures, pointing to a constant (frequency-independent) mean free path. It is often presumed that the value obtained can be identified with grain size. While this is likely valid for magnon transport, considering that the magnon origin is mostly in the nearest-neighbor exchange interactions, the phonons can traverse the boundary depending on their wavelength (see below). The observation of ideal *T* ^3^ dependence need not reflect the thermal resistance of the boundary itself but is rather related to the reduced connectivity of grains due to sample porosity.
II. More attention has been given to cases where phonon thermal conductivity at low temperatures changes toward *κ_ph_* ∼ *T* ^2^ dependence. This is observed experimentally in many ceramic materials, including the present CdCr_2_Se_4_. A special reference can be made to thorough research on nanocrystalline silicon with average grain sizes ranging from 550 to 64 nm by Wang et al.^31^ The authors have shown that the *T* ^2^ trend observed in the presence of important grain boundary scattering can be formally explained by a model in which the transmittivity of phonons (or equivalently, the effective value of their mean free path) depends on phonon frequency as ∼ω^−1^. This means that only phonons of low frequencies (long wavelengths) traverse through grain boundaries; see also the atomistic simulations of phonon transmittivity/reflectivity at grain boundaries of nanocrystalline Si and SiGe by Yang and Minnich.^30^
III. In an attempt to gain better physical insight into the scattering, the model of grain boundaries as a dense array of dislocations has been applied by Snyder and his colleagues for fitting over a broad temperature range of both the silicon data of Wang and their own data on thermoelectrical alloys.^27^ The authors have shown, with reference to earlier theoretical treatment by Klemens,^32,33^ that the low-temperature *T* ^2^ dependence can be explained as an effect of static strain imposed by dislocations on incoming phonons, leading to the ω-dependent scattering term with a magnitude given by the actual density of dislocations in the grain boundary.
IV. As far as the studies of magnon thermal conductivity are concerned, the kinetic gas formula $\kappa_{m}=\frac{1}{3}C_{m}v_{m}l_{m}$ gives the magnon contribution dependent on the energy-averaged magnon velocity *v*_*m*_ and the magnon free path *l*_*m*_. For a rough estimate of *v*_*m*_, at least for determination of its *T* ^1/2^-dependence, it is possible to consider the group velocity for thermal magnons at zero field (*E* = ℏ*ω_k_* = *k_B_T*), given as $v_{m}\left(k_{B}T\right)=\frac{d\omega_{k}}{dk}=2\sqrt{\frac{D\omega_{k}}{\hbar }}=\frac{2}{\hbar }\sqrt{Dk_{B}T}$ , while a rigorous derivation of the energy-averaged velocity gives a value 1.62 times larger. For a detailed analysis, see the work by Pan et al.^34^ In a model with temperature-independent *l*_*m*_, the magnon thermal conductivity should thus follow quadratic *T* ^2^ temperature dependence, as mentioned for the first time by McCollum et al.^35^ An open question remains regarding the transmittivity of magnons through grain boundaries. The study of various forms of YIG by Miura et al.^36^ has shown that the thermal conductivity of a single-crystalline sample at 2 K could be at least 55% ascribed to the magnon contribution. This ratio gradually diminished for the polycrystalline sample with 20 μm grains to ∼30%, and for samples with 2 and 0.5 μm grains, the magnon contribution to macroscopic heat transport was below the detection limit. This suggests that there is no or only very limited magnon penetration through grain boundaries, and, therefore, in ceramic samples, the heat is predominantly transmitted by long-wavelength or surface phonons.

### Low-Temperature Thermal Conductivity of Densified CdCr_2_Se_4_ Sample

The original material, whose properties we presented in Figure 4, was prepared with a relatively large porosity of 17%. To sort out the effects of the porosity and the grain boundary itself on the thermal conductivity, the material was subjected to densification by spark plasma sintering at 300 MPa, followed by annealing at 500 °C in a selenium atmosphere for 24 h. This resulted in the sample porosity being largely reduced to 5%. As seen in the upper and lower panels of Figure 5, the dense sample shows thermal conductivity with a significantly enhanced phonon peak and a four-times larger magnitude when compared to the porous sample. A steep drop is observed with increasing temperature, ending in an anomaly at *T*_C_ ∼ 130 K. No tendency toward the *T* ^−1^ trend can be identified. In our understanding, the reason for such behaviors lies in the coupling of lattice dynamics with ferromagnetic order in CdCr_2_Se_4_, and the observed drop in the 70−130 K range reflects a gradual increase in ferromagnetic fluctuations. Above the Curie temperature, the thermal conductivity follows the *T* ^−1/2^ trend, similar to our porous sample but with twice the magnitude. Such a seemingly analytical dependence is likely only accidental and specific to the present system in the paramagnetic state. It may result from a combination of phonon−phonon Umklapp processes, atomic displacements due to local spin correlations, and some defects associated with the microstructure of our polycrystalline sample. In this context, it is worth mentioning the first-order magnetostructural transition in CrN, which represents an extreme case of the coupled lattice dynamics and spin arrangement. This is clearly manifested in the thermal conductivity, showing an incomparably large drop when approaching the antiferromagnetic Néel temperature, followed by a practically constant or even slightly increasing trend at higher temperatures (see, e.g., Figure 6 in ref.^17^).

Behaviors of the dense and porous samples below the phonon peak are illustrated in more detail in the lower panel of Figure 5. One may note that, although the low-temperature thermal conductivity of the dense sample has increased fourfold, its suppression in a field of 13 T remained similar, pointing to a practically unchanged ratio of the magnon term to the phonon term. These results suggest that porosity influences thermal conductivity, namely in the magnetically polarized state, just as a multiplying factor. Phenomenological models anticipate a reduction with increasing porosity *p* either exponential one or as (1 − *p*)^*t*^ when percolation model is implemented.^37^ A more physical approach is to implement the effective medium theory, devised originally for the treatment of electric conduction and galvanomagnetic phenomena in inhomogeneous materials (see, e.g., ref.^38^). For the simplest model of a two-phase composite with a random distribution of spherical crystallites, the solution was presented as early as the 1950s and successfully tested on experimental data for various alloy mixtures.^39^ An analogous model has been recently used for both the electric and thermal conductivity of hot-pressed (Bi, Sb)_2_Te_3_ thermoelectrics, considering their structure as a mixture of conducting material and insulating pores.^40^ Considering all these scenarios, we may conclude that, at least in the first approximation, the conductivity is reduced with porosity linearly for small *p*. This allows us to estimate, in the absence of any real single-crystal specimen, that a hypothetical, porosity-free CdCr_2_Se_4_ sample would show thermal conductivity at least five times larger than our original porous sample (*p* = 0.17) and ∼20% larger compared to the densified one (*p* = 0.05).

Taking this fact into account, we analyze first the magnon term *κ_m_* of thermal conductivity. The lower panel of Figure 5 enables to evaluate the difference between measured *κ*_0T_ and *κ*_13T_ (including the porosity correction) as *κ_m_* = 0.055 W m^−1^ K^−1^ at 2 K. At the same time, we note that the magnon heat capacity *C*_*m*_ = 0.0537 J mol^−1^ K^−1^ (7.42 × 10^4^ J m^−3^ K^−1^), the velocity of dominant magnons in for CdCr_2_Se_4_ with spin-wave stiffness of *D* = 33.5 meV Å^2^ given by $v_{m}=1.62\frac{2}{\hbar }\sqrt{Dk_{B}T}$ = 840 $\sqrt{T}=1190$ m s^−1^ and the actual grain size of ∼1 μm give an estimate of grain-boundary-limited $\kappa_{m}=\frac{1}{3}C_{m}v_{m}l_{m}$ = 0.221 W m^−1^ K^−1^ at 2 K. This four-times larger value compared to the observed one would mean that the effective magnon path is apparently only one-quarter of the grain size, i.e., about 0.25 μm. This corroborates the assumption of no penetration of magnons through the grain boundaries. In a more realistic explanation, the present result should be related rather to a large refraction power of grain boundaries so that the spin waves are mostly localized in grains and only one-fourth of their incident heat flux is transmitted further. It is of interest that despite the complexity of heat transfer in polycrystalline CdCr_2_Se_4_, the measured magnon contribution follows the ideal *κ_m_* ∼ *T* ^2^ dependence (at least in the lowest *T* = 2−6 K range, see Figure 4b). This points to a frequency-independent reflection coefficient for incident long-wavelength magnons, the value of which (R = 75%) is much larger than commonly reported for phonon reflection at grain boundaries. In analogy to backscattering of acoustic waves at the two-media interface as applied in the theory of grain-boundary thermal resistance,^41^ we are considering that the intergrain region can be viewed as a defective and less compact medium of significantly reduced spin-wave stiffness *D’* and the grain-boundary refraction is predominantly determined by the relative change of the magnon group velocity, $R∼{\left(\frac{{v_{m}-v_{m}}^{\prime }}{v_{m}}\right)}^{2}=\frac{D-D^{\prime }}{D}$ .

The phonon part of thermal conductivity, evidenced in the lower panel of Figure 5 as κ_13T_, makes, after porosity correction, a value of *κ_ph_* 0.110 W m^−1^ K^−1^ at 2 K. Applying the kinetic gas formula $\kappa_{ph}=\frac{1}{3}C_{ph}v_{ph}l_{ph}$ with heat capacity *C*_*m*_ = 0.0072 J mol^−1^ K^−1^ (1.00 × 10^4^ J m^−3^ K^−1^) and sound velocity v = 2400 m/s, we estimate the phonon mean free path to be effectively ∼1.4 μm, which corroborates the estimated average grain size. The large difference in the mean free path between magnons and phonons suggests that magnons are much more sensitive to “structural imperfections” than phonons. This is a rather unexpected result since, based on the intrinsic scattering mechanisms and experiments on single-crystal materials, the magnon free path generally exceeds that of phonons.

The very short magnon free path we deduced above for the porosity-free CdCr_2_Se_4_ polycrystalline material (effective values *l_m_* = 0.25 μm, *l_ph_* = 1.4 μm at 2 K) can be understood by considering that, apart from magnetic defects (e.g., the presence of Cr^2+^ due to selenium vacancy V_Se_) there are random atomic displacements associated not only with grain boundaries but also with coalescing crystallites, dislocations, etc. Even if small, such atomic displacements may dramatically influence magnetic interactions, specifically in systems where competing exchange paths exist between neighboring spins, the strengths of which depend on different hopping integrals when the tight-binding model is used.^42^ As an illustrative example how orbital overlaps and, consequently, the hopping integrals vary with interatomic distances, we refer to recent study of magnetostructural transition in the CrN system possessing rock-salt structure.^3^ Similarly, the present CdCr_2_Se_4_ system is subjected to the competition between the antiferromagnetic direct Cr−Cr exchange (dependent on interatomic distance as *d*_Cr−Cr_^−10^) and the 90° ferromagnetic Cr−Se−Cr superexchange (dependent as *d*_Cr−Se_^−14^). Consequently, the dominance of ferromagnetic superexchange can locally switch to antiferromagnetic exchange, which results in strong magnon scattering.

Since phonons are shown to dominate the intergrain transport of CdCr_2_Se_4_, the observation of the porosity-independent magnon/phonon ratio of bulk thermal conductivity, (see Figure 5b for dense and porous samples) is no longer surprising. This means that the reduction with porosity need not be a result of a mere geometric factor (effective cross-section), but there can be other scattering mechanisms, frequency-dependent or independent, acting on traversing phonons. Here, in analogy to Ziman’s “fluttering” of quasi-free dislocations in single crystals, we anticipate that surface atoms of CdCr_2_Se_4_ crystallites/grains can occupy multiple sites with certain barriers between them, and under the elastic strain of incoming phonons, they are “rolling around” like pebbles at a windy shore. However, we cannot estimate, before proper quantum-mechanical treatment is available, how large the scattering effect on phonons can be. Another possible mechanism to consider, also in porosity-free polycrystals, is the dislocation strain described by Kim et al.^27^ Its strength, however, is hardly predictable for common samples that possess high-angle grain boundaries.

Finally, we are returning to the suppression of thermal conductivity in the magnetic field and the *T* ^2^ dependence of the magnon contribution obtained for the present samples. First, there is a question of whether the application of a magnetic field also influences the phonon contribution of ferromagnetic CdCr_2_Se_4_, e.g., due to the magnetization of volume impurities containing paramagnetic ions, which might bring additional stress to the main phase. Considering that the amount of foreign phases in the present samples has been found to be negligible (at most ∼1%), we argue that, at least at the lowest temperatures, the data obtained at 13 T correspond well to *κ_ph_* at zero field. With an increase in temperature to ∼8 K, the situation becomes more complicated. As illustrated in the inset of Figure 4a, the *κ*_0T_−*κ*_13T_ data, even after correction for incomplete reduction of magnons, cease to follow the expected *T* ^2^ trend of *κ_m_*. There is also some change in the character of isothermal *κ_m_* (*H*, *T* ≥ 10 K) curves, where instead of the theoretically predicted gradual quenching exemplified in Figure 4b for 2.5 K and scaled as *H/T*, a certain drop in magnon thermal conductivity has been observed in low fields, followed by a nearly constant value in the range of 1−13 T.^7^ First, let us note that the *T* ^2.3^ dependence of phonon thermal conductivity suggests that the *l_ph_* for our porous sample is effectively reduced with temperature as *T* ^−0.7^. This predicts its decrease from 1.4 μm at 2 K to ∼0.5 μm at 8 K, while for the dense sample (*T* ^2.5^ dependence), a slower decrease is anticipated but still below the grain size. At the same temperature (∼8 K), the velocity of dominant magnons reaches that of acoustic phonons (sound velocity of 2400 m/s). One may thus expect the onset of some phonon-magnon scattering, and in our opinion, the applied magnetic field brings two opposing effects which can explain the peculiar behavior of *κ*_0T_−*κ*_13T_ above 8 K. In particular, we consider that the predicted suppression of magnon conductivity due to the external magnetic field can be partially compensated by an increase in lattice contribution due to decreased phonon scattering on magnons. With increasing temperatures above ∼20 K, we expect that the effect of phonon-magnon scattering becomes gradually unimportant since the magnon population and their contribution to thermal conductivity, though increasing, are overwhelmed by a still steeper increase and dominance of the phonon contribution.

## Conclusion

The present study focused on the thermal conductivity of the ferromagnetic thiospinel CdCr_2_Se_4_. Two polycrystalline samples with different porosities were investigated − the first one prepared by standard sintering (porosity 17%) and the second one densified by the spark plasma procedure (porosity 5%). The thermal conductivity experiments were performed in the range from room temperature down to 2 K. To distinguish the role of phonons (standard lattice contribution) from that of magnons, additional measurements in external magnetic fields up to 13 T were conducted below 20 K. The results were discussed with reference to older and recent literature sources describing numerous scattering mechanisms that pertain to both intrinsic (phonon−phonon, phonon−magnon) processes and defect/microstructure-related extrinsic ones.

As a noteworthy and rather unexpected result, it can be mentioned that the mean free path for magnons at the lowest measured temperatures is well below the CdCr_2_Se_4_ grain size. Such an observation is related to the presence of local spin fluctuations arising due to small variations in interatomic distances. The reason for such strong coupling between the spin and lattice degrees of freedom lies in a competition between the antiferromagnetic Cr−Cr direct exchange and the 90° ferromagnetic Cr−Se−Cr superexchange, each having a specific dependence on local atomic displacements. On the other hand, the mean free path of phonons is about four times larger and seems to be limited by grain-boundary scattering. Due to its slight decrease with increasing temperature, the contribution to the low-temperature thermal conductivity declines from the ideal *T* ^3^ trend to *κ_ph_* ∼ *T* ^2.5^ for the dense sample and ∼*T* ^2.3^ for the porous one.

Another specific feature is the behavior of thermal conductivity above the phonon peak at ∼50 K. Instead of the commonly observed *T* ^−1^ decrease, there is a much steeper drop ending with an anomaly at the ferromagnetic *T*_C_ ∼ 130 K. With further temperature increase up to ∼300 K, the thermal conductivity decreases only slightly, following an apparent *T* ^−0.5^ trend. The behaviors mentioned point again to a strong coupling between the lattice dynamics and spin states. It is obvious that the spin fluctuations approaching *T*_C_ and local spin correlations surviving above *T*_C_ significantly influence the temperature dependence of thermal conductivity in CdCr_2_Se_4_.

## Supplementary Material



## Data Availability

Data associated with this study are available from Zenodo at doi.org/10.5281/zenodo.18622579.

## Notes

Fig. S1. Powder X-ray diffraction patterns of the CdCr_2_Se_4_ sample prepared via SPS; Fig. S2. Powder X-ray diffraction patterns of the CdCr_2_Se_4_ sample prepared via hot-pressing; Fig. S3. SEM images (secondary electrons) of the samples; Fig. S4. SEM images (backscattered electrons from polished surface) of the samples; Fig. S5. SEM image and elemental X-ray maps of the selenized SPS sintered “dense” sample; Table S1. Energy-dispersive X-ray analysis of several points on the dense sample (PDF)
