# Supplementary material for: Thermal Conductivity of CdCr2Se4 Ferromagnet at Low Temperatures: Role of Grain Boundaries and Porosity
Source: Langmuir. 2026 Mar 18;42(12):8741–9. doi: 10.1021/acs.langmuir.5c06870 (PMC13045008; doi:10.1021/acs.langmuir.5c06870)
Supplement: Supplementary file 1 [file la5c06870_si_001.pdf]

Supporting information

Thermal conductivity of $CdCr_2Se_4$ ferromagnet at low temperatures: role of grain boundaries and porosity

Jiří Hejtmánek[1*], Kyo-Hoon Ahn[1], Zdeněk Jirák[1], Petr Levinský[1], Jiří Navrátil[1], Sandy Al Bacha[2], Emmanuel Guilmeau[2] and Karel Knížek[1]

[1]Institute of Physics of the Czech Academy of Sciences, 162 00 Prague 6, Czech Republic
**Corresponding author: hejtman@fzu.cz*

[2]CRISMAT, CNRS, Normandie Univ, ENSICAEN, UNICAEN, 14000 Caen, France

Keywords: $CdCr_2Se_4$; specific heat capacity; thermal conductivity; magnons; ferromagnetic materials; porous ceramics; grain boundaries.

Number of pages: 5

Number of figures: 5

Number of tables: 1

Table of Contents

Fig. S1. Powder X-ray diffraction patterns of the $CdCr_2Se_4$ sample prepared via SPS S 2

Fig. S2. Powder X-ray diffraction patterns of the $CdCr_2Se_4$ sample prepared via hot-pressing S 3

Fig. S3. SEM images (secondary electrons) of the samples S 4

Fig. S4. SEM images (backscattered electrons from polished surface) of the samples S 4

Fig. S5. SEM image and elemental X-ray maps of the selenized SPS sintered “dense” sample S 5

Table S1. Energy-dispersive X-ray analysis of several points on the dense sample S 5

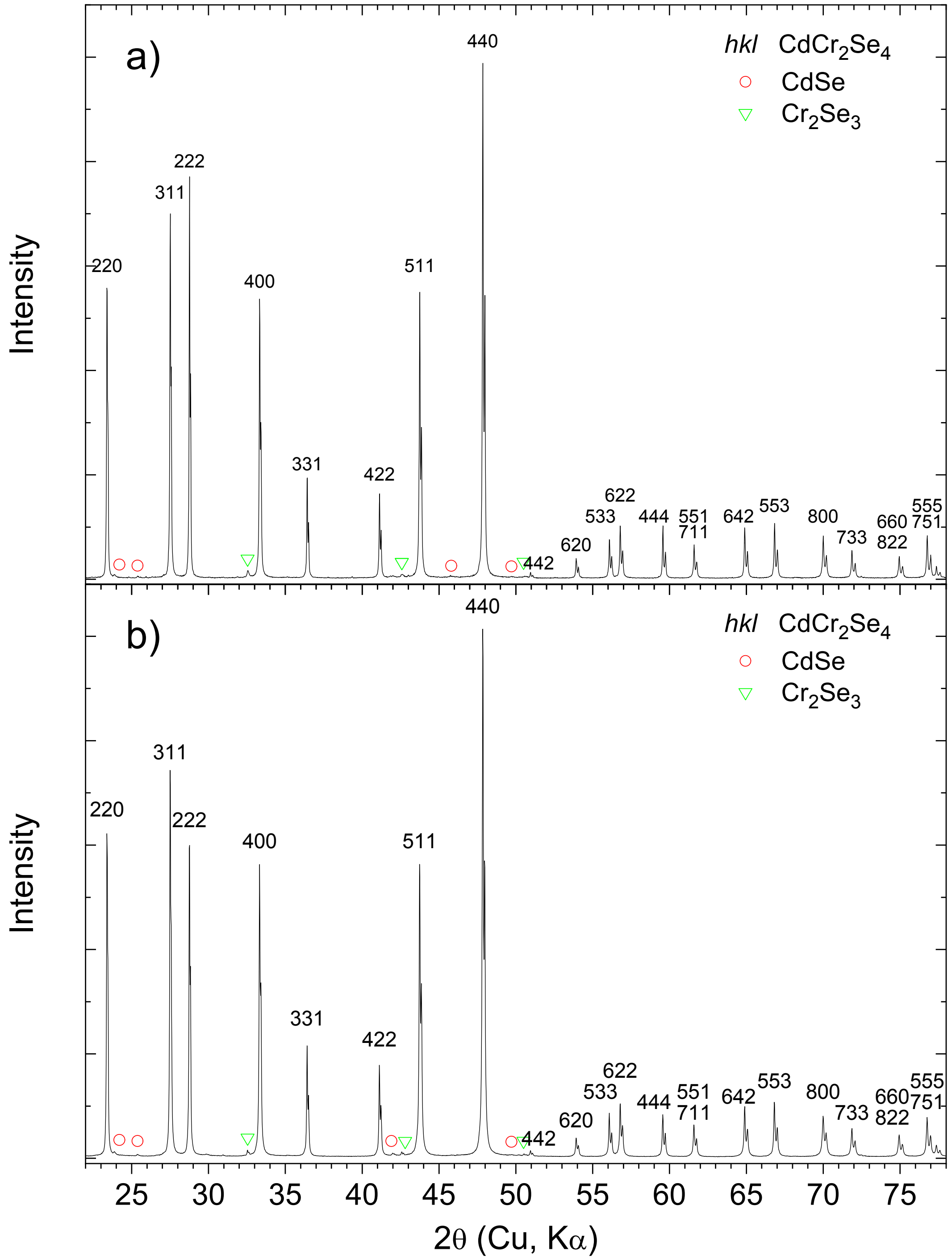

Fig. S1. Powder X-ray diffraction patterns of $CdCr_2Se_4$ samples, a) before SPS sintering, b) after SPS sintering and selenization (i.e. the "dense" sample). The *hkl* indexes of the main reflections and main peaks of impurity phases are indicated. The volume amount of each minority phase, CdSe (o) and Cr_2Se_3 (∇), is less than 1%.

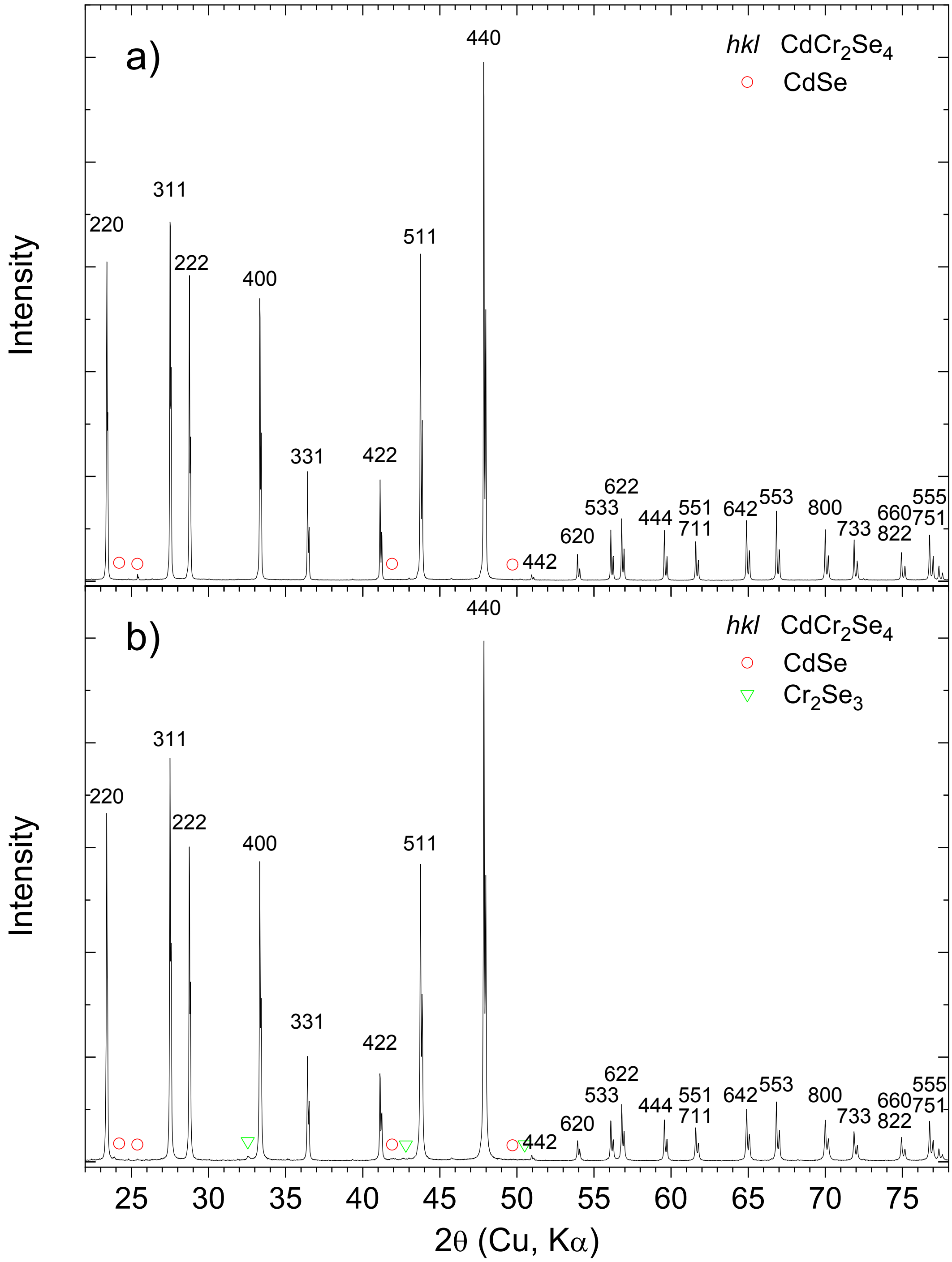

Fig. S2. Powder X-ray diffraction pattern of the hot-pressed (HP) "porous" $CdCr_2Se_4$ sample. a) powder sample before HP sintering. b) solid sample after HP sintering. The *hkl* indexes of the main reflections and main peaks of impurity phases are indicated. The volume amount of each minority phase, CdSe (o) and Cr_2Se_3 (∇), is less than 1%.

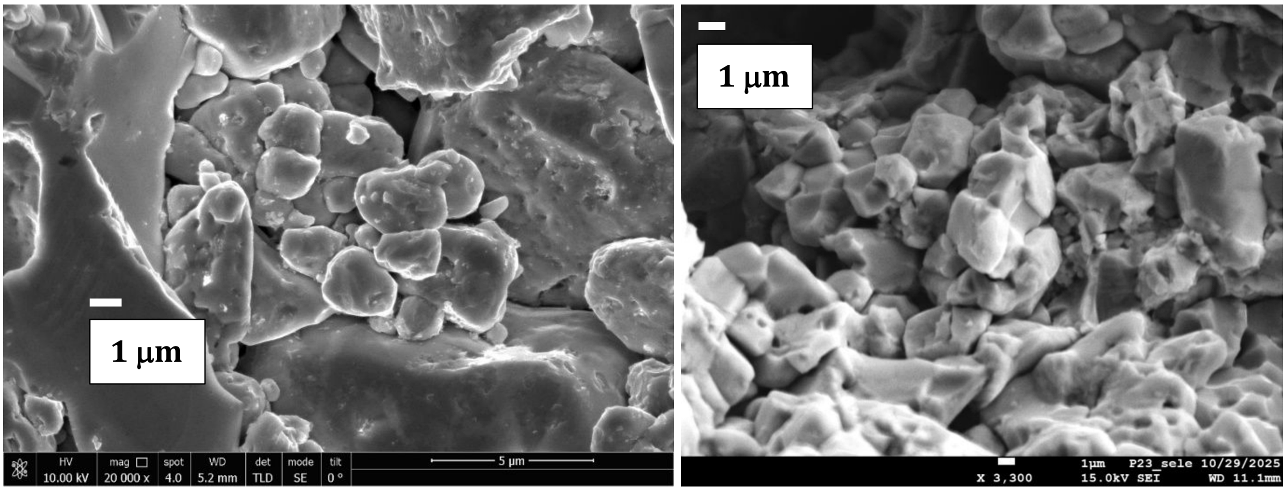

Fig. S3. Scanning electron microscope images (secondary electrons) of the samples. Left: hot-pressed porous sample (83% density), right: SPS sintered ceramics with post annealing in selenium vapor, i.e. the dense sample (95% density).

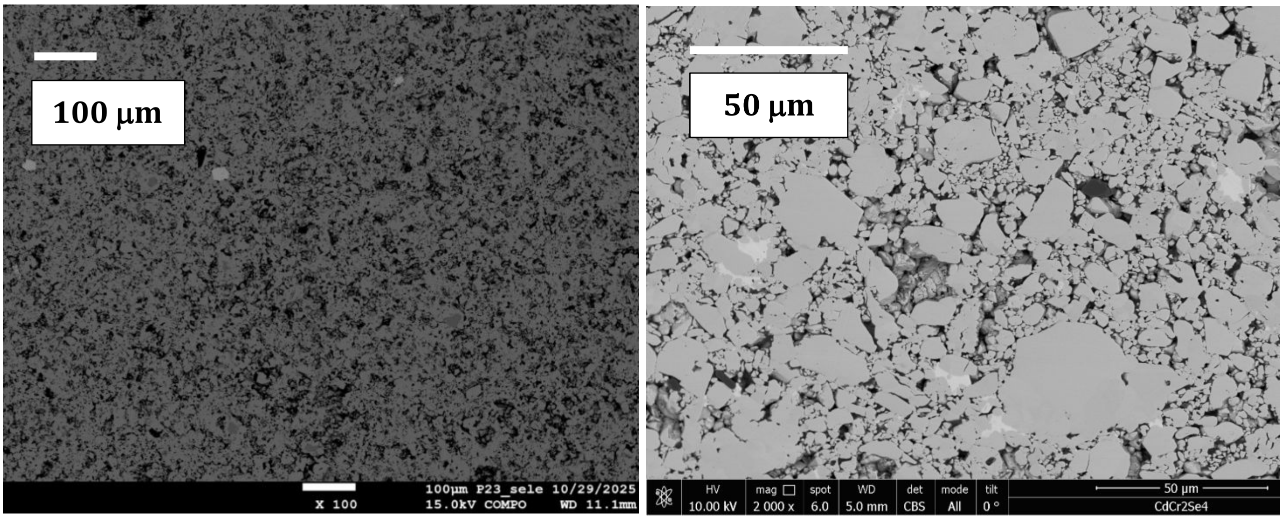

Fig. S4. Scanning electron microscope images (backscattered electrons from polished surface). Left: hot-pressed porous sample (83% density), right: SPS sintered ceramics with post annealing in selenium vapor, i.e. the dense sample (95% density).

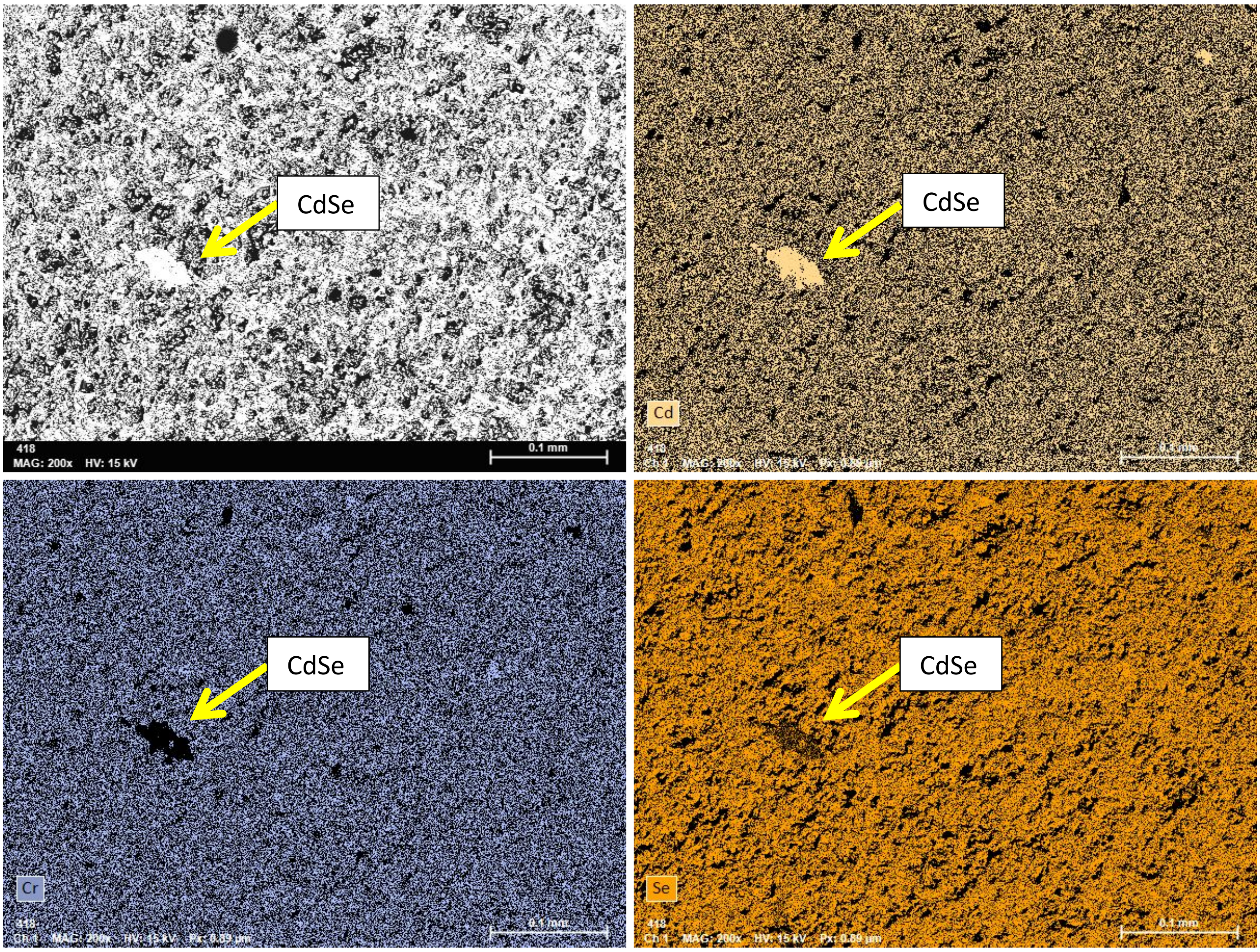

Fig. S5. Scanning electron microscope image and elemental X-ray maps of the selenized SPS sintered “dense” sample. The morphology of CdSe impurities, represented by large, isolated grains, guarantees their negligible role in thermal conductivity which is of “percolative” character. The Cr_2Se_3 impurities are represented by similar, spatially isolated grains with the size of units of micrometers.

Table S1. Energy-dispersive X-ray analysis of several points on the dense sample. The data was collected using the Jeol JXA-8230 instrument equipped with a Bruker QUANTAX 200 spectrometer. Quantitative analysis was performed with the method PB-ZAF.

Atomic %					Normalized to 7 atoms			
Cd	Cr	Se	sum		Cd	Cr	Se	
14.50	28.06	57.44	100.00		1.02	1.96	4.02	
14.80	28.00	57.20	100.00		1.04	1.96	4.00	
14.90	28.38	56.73	100.01		1.04	1.99	3.97	
14.01	29.90	56.09	100.00		0.98	2.09	3.93	
14.12	29.83	56.05	100.00		0.99	2.09	3.92	
					**1.01**	**2.02**	**3.97**	mean
					0.02	0.06	0.04	±
